# Identifying Distinct Tourette Disorder Subtypes using Clinical Data

**DOI:** 10.1101/2025.11.09.25339700

**Published:** 2025-11-11

**Authors:** Subramanian Krishnamurthy, Tourette International Collaborative Genetics (TIC Genetics), Robert A. King, Jay A. Tischfield, Gary A. Heiman, Jinchuan Xing

**Affiliations:** 1 Department of Genetics, Rutgers, The State University of New Jersey, Piscataway, NJ 08854, USA; 2 Human Genetics Institute of New Jersey, Rutgers, The State University of New Jersey, Piscataway, NJ 08854, USA; 3 Yale Child Study Center, Yale School of Medicine, New Haven, CT 06510, USA

## Abstract

**Background:**

Tourette Disorder (TD), or Tourette Syndrome, is a highly heterogeneous childhood-onset neurodevelopmental disorder with a global prevalence of 0.5%. TD’s large phenotypic heterogeneity, variable heritability patterns, and frequent but varied comorbidity with other neurodevelopmental disorders indicate that different TD patients might have different etiologies.

**Objectives:**

In this study, we performed unsupervised clustering of clinical data, such as categorical diagnoses and comorbidities, from 865 subjects with TD from the Tourette International Collaborative Genetics (TIC Genetics) study to detect Tourette Disorder subtypes.

**Methods:**

We used two different clustering methods, K-Means and Bayesian Hierarchical Clustering to detect phenotypic subtypes.

**Results:**

We identified five distinct, clinically relevant subtypes. These subtypes are characterized by both previously described TD comorbidities, such as Obsessive-Compulsive Disorder (OCD) and Attention Deficit/Hyperactivity Disorder (ADHD), as well as other characteristics, such as sex and region.

**Conclusions:**

Defining clinically relevant TD subtypes could enable better diagnostics, treatment, and the understanding of TD etiology. In addition, stratified analysis of genetic data based on these phenotypic subtypes may help identify genes contributing to each TD subtype and provide insights into the disease variability.

## Introduction

Tourette Disorder (TD), also called Tourette Syndrome, is characterized by multiple motor tics and at least one vocal tic, occurring many times a day (usually in bouts) nearly daily or intermittently for at least a year, with an onset before age 18 [1]. Most subjects with TD over the age of 10 report premonitory urges that are relieved by tics [2]. Coprophenomena (such as copropraxia or coprolalia) may occur when tics are most severe [2]. In addition, there are also sex-dependent differences in the phenotypic expression of TD, with males more often showing tic-related behaviors and females more often exhibiting Obsessive-Compulsive Symptoms/Behaviors (OCS/OCB) [3, 4]. Twin and family studies have established that TD bears a strong genetic component with an unclear inheritance pattern, probably due to polygenic cause [5, 6]. Large-scale genetic studies of TD have identified specific classes of genetic variation that contribute to the genetic risk of TD, including common variants [7], segregating variants, rare copy number variants, and *de novo* variants that impact protein-coding sequences [8–11].

Approximately 88% of TD subjects have one or more other Neuro Developmental Disorders (NDDs) as comorbidities [12], such as Obsessive-Compulsive Disorder (OCD, ~30–50%), Attention Deficit/Hyperactivity Disorder (ADHD, ~50–60%) and to a lesser extent, Autism Spectrum Disorder (ASD, ~10%), as well as anxiety, depression, and other conditions [13]. Given the substantial genetic and phenotypic heterogeneity of TD, identifying distinct subtypes could assist in better diagnosis and treatment, as well as enable a better understanding of genetic architecture through stratified analysis of genetic data.

Several prior studies (reviewed in [14]) have attempted to identify common factors and subtypes of TD. For example, one study examined heritability and polygenic load associated with TD, OCD, and ADHD using symptom-level factors [15]. Latent class analyses of subjects with TD identified three major TD-affected groups: 1) TD + OCS/OCB, 2) TD + OCD, and 3) TD + OCD + ADHD [16]. In two studies of TD comorbid disorder clusters and their heritability, TD + OCD and TD + OCD + ADHD were considered heritable while TD + ADHD was not [17, 18]. In another study of clinical factors of 139 subjects with TD, two subtypes of tic disorders with severe (21.6%) and mild (78.4%) symptoms were identified [19]. A 6-factor exploratory factor analysis of forty-nine motor and phonic tics in 3,494 individuals (1,191 TS probands and 2,303 first-degree relatives) identified social disinhibition as a heritable subtype of TD [20].

Although these studies provided useful information, they had limited ability to comprehensively identify TD subtypes, because many of them were based on cohorts with either limited subsets of available phenotypic information (i.e., comorbidities or symptoms), small sample size, and/or subjects that were demographically homogeneous. In this study, we identified subsets with TD using unsupervised clustering of clinical and demographic data from the Tourette International Collaborative Genetics (TIC Genetics) study, a large international study with over 20 sites across the United States, Europe, Israel, and South Korea [21]. This large, uniformly assessed clinical dataset enabled us to identify five clinically relevant subtypes that will enhance the understanding of the heterogeneity of TD.

## Materials and Methods

### Clinical Data

The clinical data were collected between 2011 and 2023 as part of the TIC Genetics Study [21]. The Institutional Review Board approved the study protocol at each local site. Informed consent was obtained from all participants (or in the case of minors, from their parents). The clinical assessment methods and definitions of TD, OCD, ADHD, and Trichotillomania have been previously described in detail [21], and are based on the Diagnostic and Statistical Manual of Mental Disorders—Fourth edition, Text Revision (DSM-IV-TR) [22] or Fifth edition (DSM-5) [1]. Clinicians were trained to record all symptoms and perform diagnoses in a consistent manner to ensure quality and reliability across all sites.

Detailed clinical data were acquired from an extensive written questionnaire which was completed by the participant (or the parent in the case of young probands); this information was validated in a semi-structured interview by an experienced clinician and recorded in a standardized fashion. Items included demographic information, details of tic, OCS/OCB/OCD, ADHD, and trichotillomania (including ages of onset), and other potentially relevant conditions. Based on the questionnaire and interview information, the clinician made lifetime diagnoses for 4,435 subjects, including probands with TD, both affected and unaffected parents, and some affected and unaffected siblings [21].

### Data Preprocessing

To improve the precision of subtype identification, we excluded other tic disorders, such as other chronic, provisional, and transient forms. Among subjects that had TD, we excluded those that have any missing or “unable to rate” value for any of the Tic, Obsessive Compulsive, or Attention-disorder-Hyperactivity diagnoses. We also excluded subjects with potentially confounding factors flagged by clinicians, including “atypical presentation”, “psychosis”, “other severe neurological condition”, “congenital anomalies”, “genetic syndrome/chromosomal abnormality”, “other significant psychiatric history”, and “other significant medical history”. From each family, we selected only one member diagnosed with TD. 22 centers around the world were grouped into 3 regions: USA, Europe, and Asia. A description of the detailed clinical data variables for each subject included in this study is available in Supplementary Table S1.

Some parameters in our clinical dataset are mutually exclusive. For example, for OCD diagnosis, each subject belongs to one of the following categories: No OC disorder/symptoms, OC Subclinical, OC Symptoms, or OCD. In these cases, these mutually exclusive parameters were combined into a single categorical parameter (Supplementary Table S2). Age of onset for a given disorder was converted into two categories: “Early” (<= 10y) and “Late” (> 10y). In a few cases (<10%) where the age of onset was not available, age at diagnosis was used as a proxy. The age of onset of subjects with no diagnosis of the disorder was coded as “Never”. We used two categories for birth: “single” or “multiple” (>=2). After preprocessing, 14 categorical parameters were selected for clustering (Supplementary Table S2).

### Exploratory data analysis and clustering method testing

Characteristics and distribution of individual parameters were analyzed to examine the dataset. Pairwise correlations between parameters were calculated as Cramer’s V statistics using the “CramersV” function in the R package “rcompanion” (version 2.5) [23]. Several clustering methods were tested (Supplementary Table S3), and two unsupervised clustering methods, K-Means and Bayesian Hierarchical Clustering (BHC) [24], were selected to identify TD subtypes. As the statistical methods chosen for our study are designed to handle highly correlated parameters, we retained the correlated parameters to provide additional insight into specific cluster characteristics.

### K-Means clustering

K-means clustering is an unsupervised clustering method that partitions n observations into k clusters in which each observation belongs to the cluster with the closest cluster mean. Multiple Correspondence Analysis (MCA) [25] was used to convert categorical parameters into principal components (PCs). This enabled us to: 1. reduce noise by removing less significant PCs with small contribution to the overall variance, and 2. use clustering methods that require continuous variables. K-means clustering was performed using the K-Means function in R using the “stats” package (version 4.4.3). The number of clusters (centers = k) from 2 to 20 were tested. To ensure robust convergence and reproducibility, the algorithm was run with 5,000 iterations and 1,000 random starts.

Euclidean distance was used as the distance metric and the clustering method described by Hartigan and Wong (1979) [26]. The optimal number of clusters was identified based on the silhouette statistics (maximal inter-cluster / intra-cluster mean distance).

### Bayesian Hierarchical Clustering (BHC)

The BHC method performs bottom-up hierarchical clustering, using a Dirichlet Process (infinite mixture) to model uncertainty in the data and Bayesian model selection, to identify clusters that can be merged at each step [24]. This method works with categorical data and optimizes hyperparameters to infer the optimal number of clusters. BHC clustering was performed using the BHC BioConductor package (version 1.56.0) in R with default parameters.

### Cluster characteristics

Parameter importance was inferred using V-measure (or Normalized Mutual Information, calculated by “V-test” using “FactoMineR”, version 2.11, in R). V-measure is a single metric, computed based on percentage of the cluster that has a given characteristic and percentage of the population with a given characteristic that is in the cluster, which is an average of homogeneity and completeness. Key parameters that define the cluster characteristics have high V-measure. The relative importance of each parameter on each cluster was determined by Fisher’s exact test or V-Test.

### Tools

R was used for all statistical analyses, and an overview of all R packages used in the analysis is listed in Supplementary Table S4.

### Data availability

All data used in this study are provided in Supplementary Table S1.

## Results

### Data pre-processing and exploratory analyses

**Figure 1.**
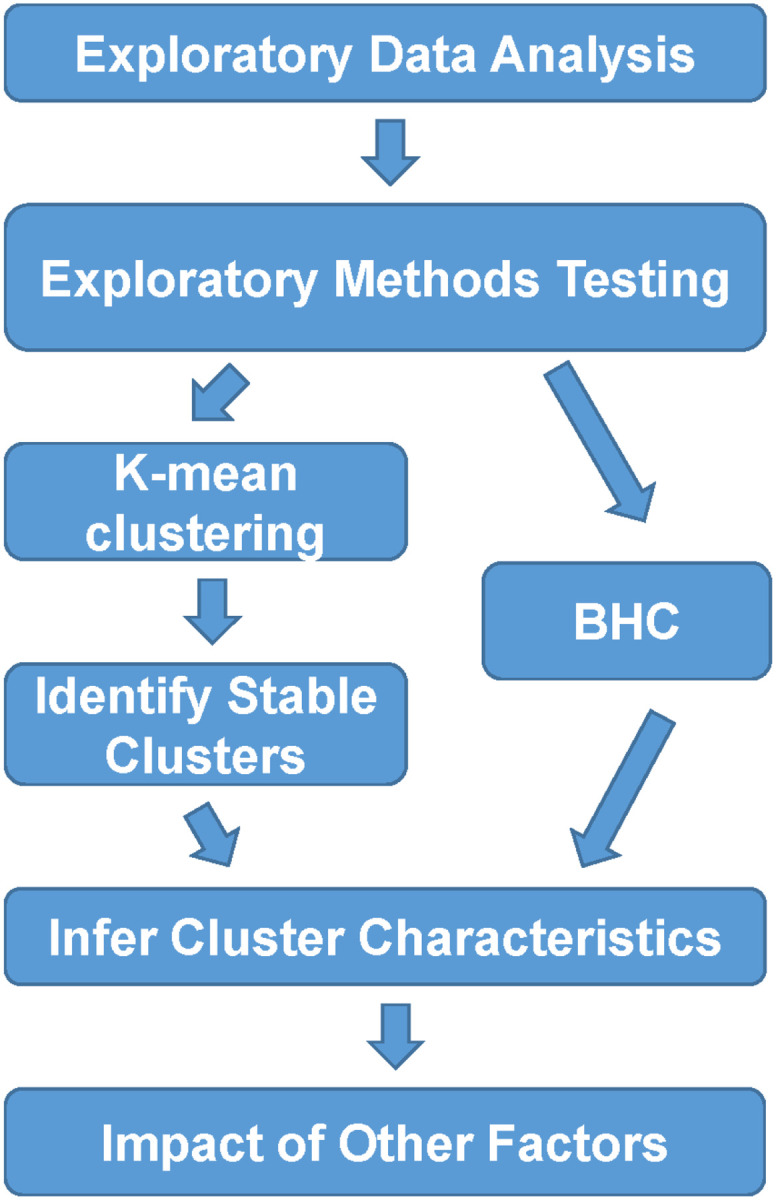
Analysis Pipeline overview. We first performed exploratory data analysis to understand the characteristics of data, such as parameter distribution, completeness, and correlation. We then evaluated several unsupervised clustering methods (Supplementary Table S3) to identify methods that produce stable clusters. After testing, we selected two methods, K-Means and BHC for the final clustering and analysis of the data. After the cluster identification, we used V-Test to identify key factors that define the characteristics of each cluster and Fisher’s exact test to assess the impact of factors on each cluster.

An overview of the data processing and analysis pipeline is provided in Figure 1. For clustering, we selected subjects diagnosed with TD (1,548) and excluded subjects with genetic abnormalities or severe psychiatric conditions (421) (see Methods for details). In addition, we excluded subjects with who were rated “Unable to rate” for Tic, Obsessive-compulsive, or Attention-deficit-Hyperactivity disorder diagnoses. To limit the effects of genetic relatedness, we selected one offspring from each family. The final data set used for identifying subtypes includes 865 offspring diagnosed with TD. Counts by category for each parameter are included in the Supplementary Table S2.

To understand the relationship between selected parameters, we computed pairwise correlations among the parameters using Cramer’s V statistic (Supplemental Figure 1). The observed high correlations between OCD-related parameters, ADHD-related parameters, and Trichotillomania-related parameters are expected. There were no other significant correlations among the parameters.

After evaluating eight unsupervised clustering methods (Supplementary Table S3), and many different subsets of data (Supplementary Table S5), we selected two methods that produced stable clusters with our dataset: K-Means and Bayesian Hierarchical Clustering (BHC). Because these methods can accommodate correlated parameters, we included correlated parameters such as disease diagnosis, subtypes, and current symptom, in our analyses.

### K-Means Clustering

To perform K-Means clustering, we used MCA to generate PCs from the dataset. When we provided all PCs to K-means clustering in our initial testing, it resulted in unstable clusters. These results are probably due to inherent variability in the clinical data. The later PCs capture mostly noise rather than meaningful structure in the clinical data. Therefore, we evaluated systematically excluding the least important PCs, one at a time, to reduce the noise in the dataset. For each set of PCs, we assessed the cluster number (K) from 2 to 20 and repeated the analysis 100 times to assess the stability. This resulted in the selection of the top three PCs (account for >35% of the variance), that produced five stable clusters (Silhouette Plot, Supplementary Figure 2).

**Figure 2.**
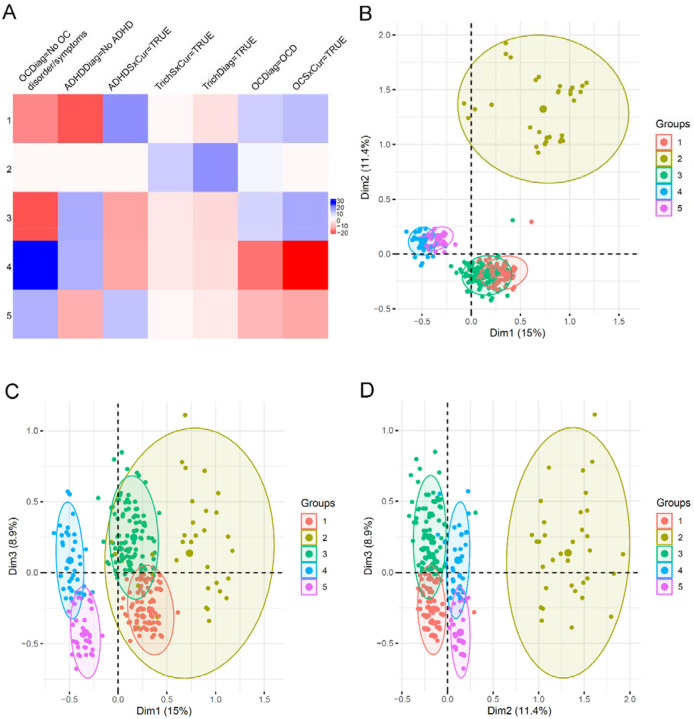
K-Means Clusters. **A.** Major factors determining cluster characteristics. Positive values are positive correlations, while negative values are negative correlations. The larger the value, the stronger the correlation. **B-D**. Cluster plots on first three principal components.

Next, we identified the parameters that most significantly correlated (positively or negatively) with cluster identity (Figure 2A). For example, trichotillomania diagnosis is positively correlated only with cluster 2 (TrichDiag=TRUE), indicating grouping of subjects with trichotillomania in this cluster. ADHD is negatively correlated with cluster 3 (ADHDDiag=No ADHD). Plotting cluster memberships along the top three PCs confirmed clear separation of clusters along the three PCs, suggesting robust clustering and visually confirming the quality of clusters identified (Figures 2B–D). .

The detailed contribution and significance of each parameter to each cluster are listed in Supplementary Data: K-Means.xlsx, including percentage of subjects in a category in each cluster, percentage of subjects in each cluster in a category, percentage of subjects in a category in the whole data set, and v-test for the significance of each parameter. These results provide details for each statistically significant parameter for each cluster, allowing us to fully understand the cluster characteristics. Overall, 12 out of 14 parameters show statistically significant contribution to the clusters, including all parameters associated with OCD, ADHD, trichotillomania, region, and sex (Supplementary Data: KMeans.xlsx – test.ch2 tab).

Based on the parameter contribution to the clusters and the individual membership of each cluster, we determined the defining characteristics of each cluster (Supplementary Table S6). To make the results below more comprehensible, we describe all diagnosis parameters for a given disorder as one category in the sections below (e.g., “ADHD” for ADHD diagnosis and ADHD current symptom) and included the p-value for the most statistically significant parameter.

Cluster 1 subjects (n=210) have ADHD (3.6E^−71^), early OCD age of onset (1.7E^−46^), no trichotillomania (p=1.1E^−5^), higher prevalence of males (2.2E^−8^) and ASD (p=7.9E^−4^). Cluster 2 subjects (n=40) have trichotillomania (p=6.7E^−70^), with higher prevalence of subjects from the USA (p=6.9E^−4^) and OCD (p=8.0E^−4^). Cluster 3 subjects (n=289) have high prevalence of OCD (p=4.0E^−52^), low levels of ADHD (p=4.0E^−49^), no trichotillomania (p=5.4E^−8^), higher prevalence of females (1.1E^−6^) and European subjects (1.8E^−3^). Cluster 4 subjects (n=229) have low NDD comorbidities, with no OCD (p=7.4E^−127^), very low ADHD (p=7.4E^−45^), no trichotillomania (p=3.3E^−6^), low prevalence of ASD (p=5.1E^−6^), and a high proportion of South Korean subjects (2.3E^−6^). Cluster 5 (n=97) comprises subjects with TD that have no OCD (p=5.6E^−45^), high prevalence of ADHD (p=2.6E^−32^), ASD (p=1.0E^−3^), and males (p=1.1E^−4^).

### BHC

**Figure 3.**
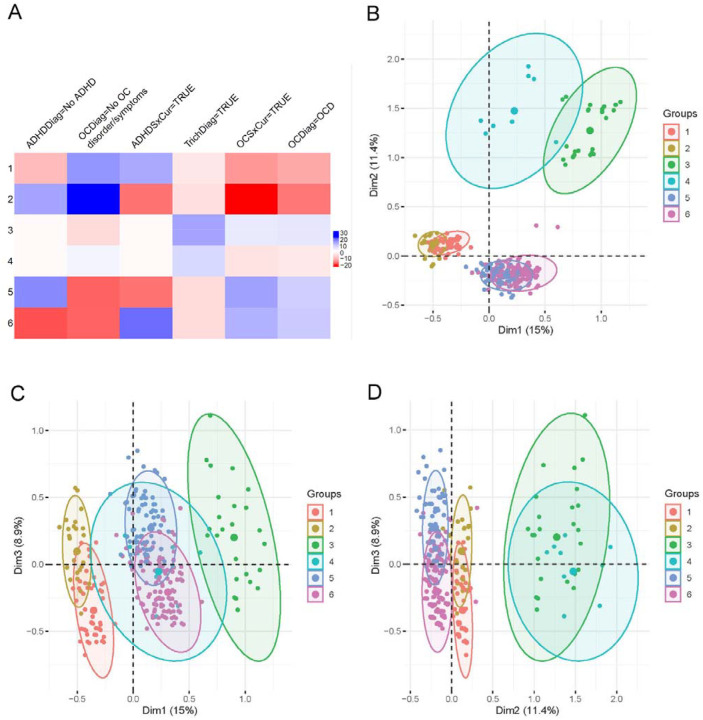
BHC Clusters. **A.** Cluster characteristics heatmap. Positive values are positive correlations, while negative values are negative correlations. The larger the value, the stronger the correlation. **B-D**. Cluster plots on first three principal components.

BHC clustering using the data set with 14 categorical parameters identified six clusters. We identified the parameters that most significantly correlated (positively or negatively) with cluster identity (Figure 3A). Plotting cluster memberships along the top three PCs confirmed clear separation, suggesting robust clustering (Figures 3B–D) and similarity between clustering produced by K-Means and BHC. The detailed contribution and significance of each parameter to each cluster are listed in Supplementary Data BHC.xlsx. Like the K-mean clustering analysis, 11 out of 14 parameters show a statistically significant contribution to the clusters (Supplementary Data: BHC.xlsx – test.ch2 tab).

Next, we examined the characteristics of each of the six clusters (Supplementary Table S7). Cluster 1 (n=120) comprises subjects with TD that have a high prevalence of ADHD (p=8E^−44^), no OCD (p=5.2E^−57^), and no trichotillomania (p=2.2E^−3^). Cluster 2 subjects (n=206) have low NDD comorbidities such as no OCD (p=7.9E^−110^), very low ADHD (p=7.3E^−60^), and an absence of trichotillomania (p=1.4E^−5^), and a low prevalence of ASD (p=3.6E^−5^). Cluster 3 subjects (n=30) have trichotillomania (p=2.9E^−47^), with a higher prevalence of OCD (p=2.8E^−8^), and are more likely to be from the USA (1.2E^−3^). Cluster 4 subjects (n=10) have trichotillomania (p=1.4E^−14^) and no OCD (p=1.3E^−3^). Cluster 5 subjects (n=250) have a high prevalence of OCD (p=2.1E^−49^), low levels of ADHD (p=2.3E^−64^), an absence of trichotillomania (p=8.1E^−7^), and an increased proportion of females (4.2E^−5^). Cluster 6 subjects (n=249) have ADHD (3.4E^−81^), OCD (1.3E^−42^), no trichotillomania (p=8.7E^−7^), and a higher proportion of males (3.6E^−5^).

### Comparison of K-Means and BHC’s results

**Figure 4.**
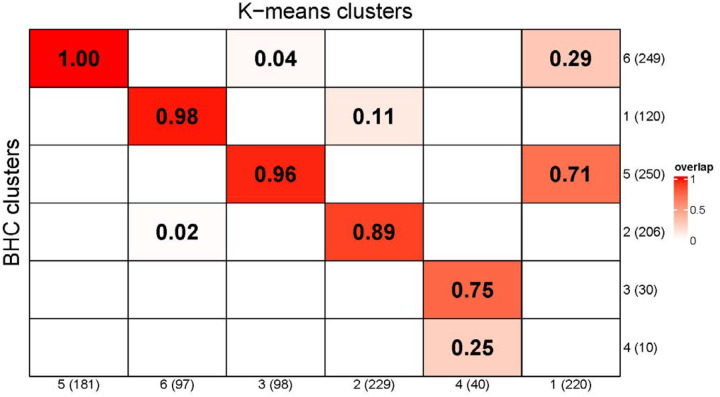
**K-Means and BHC Cluster Comparison** – Fraction of K-Means cluster members in each BHC Cluster in descending order of overlap. For example, 75% and 25% of the K-Means cluster 2 members are in BHC cluster 3 and 4, respectively.

K-Means uses a distance metric (Euclidean distance) and requires specification of the number of clusters. On the other hand, BHC uses an infinite mixture model to infer the optimal number of clusters and does not require specification of a distance metric. Fraction of members in each K-Means cluster that are in each BHC cluster is depicted in a heatmap in Figure 4. Both methods resulted in four large clusters of similar sizes and characteristics, as illustrated in PC plots (Figures 2 and 3) and Table 1. These large clusters differ from each other only in minor characteristics and slightly varying memberships. Besides the four large clusters, K-Means clustered all trichotillomania patients together in one cluster, while BHC split them into two clusters (with and without OCD). Most significant parameters (low p-value) that characterize each subtype and differentiate it from others are highlighted (Table 1).

## Discussion

Tourette disorder has high phenotypic heterogeneity, high comorbidity with other NDDs, and varying rates of shared heritability with other NDDs, suggesting distinct underlying genetic causes for different TD subjects. In this study, we performed unsupervised clustering analysis of clinical data from 865 TD subjects in the TIC Genetics study. The analyses identified five clinically relevant subtypes and highlighted shared characteristics among TD patients. Prior attempts to identify specific genes or alleles contributing to disease risk have yielded only two genes with rare damaging variants that confer large effects and a single common variant of small individual effect [8, 9]. Stratified genetic analysis of TD subjects based on additional clinical features may help identify more genetically homogenous cohorts and improve power for gene discovery. Furthermore, identifying clinically relevant subtypes could enable better diagnostics and treatment. Earlier attempts to identify subtypes often relied on small sample sets from single cohort [2, 16, 28, 29]. In addition, these studies usually relied on prior hypotheses regarding a subset of factors that were the cause of differentiation. To overcome these limitations, here we performed unsupervised clustering analyses, using consistent clinical data from over 20 global sites, without filtering parameters using prior hypothesis. This analysis enabled us to build on prior knowledge and identify novel TD subtypes.

A major challenge in clinical data analysis lies in the presence of noise and occasional misclassification, which can compromise the reliability of findings. Therefore, data selection and cleanup are essential. In addition, we also evaluated several unsupervised clustering methods to identify the optimal methods for our data set (Supplementary Table S3). After evaluating a variety of data set selection criteria and clustering methods, we selected only TD subjects for our final dataset (Supplementary Table S1) and identified two methods, K-Means and BHC, that worked well with our dataset. Final dataset was selected to be as complete and homogeneous as possible and the methods were selected to ensure stable clustering.

The two methods are quite different from each other. K-Means clustering was performed with PCs from MCA using the Euclidean Distance metric. The number of clusters was determined using Silhouette Statistics, coupled with the stability of the clusters generated. On the other hand, BHC differs from traditional distance-based agglomerative clustering algorithms in several ways: (1) It defines a probabilistic model to compute the probability of a subject belonging to any of the existing clusters in the tree; (2) it uses a model-based criterion to decide on merging clusters rather than an ad-hoc distance metric; and (3) the number of clusters is automatically determined [24]. In our analysis, K-means identified five clusters while BHC identified six clusters. Interestingly, the largest four clusters from both methods have similar size and characteristics. Because BHC used all the (categorical) data, while K-Means clustering used the first three PCs, this might account for the slight differences in cluster sizes and minor characteristics. Nevertheless, as we started without any assumption regarding the number of subtypes, the identification of similar sets of clinically relevant clusters by two different methods (Table 1) increases our confidence in the results. In addition to confirming earlier findings on the importance of highly prevalent comorbidities such as ADHD and OCD [16], this large and diverse dataset identified new clusters that illustrate the importance of other clinically relevant factors such as trichotillomania and sex in TD subjects.

Trichotillomania is an often debilitating psychiatric condition characterized by recurrent hair-pulling, which can lead to disfiguring hair loss and significant distress and social impairment, and has been associated with elevated rates of anxiety, depression, ADHD, and OCD (DSM-5) [1]. In DSM-5, it is classified under OCD-related Disorders. The condition has significant heritability according to twin studies, a shared genetic liability with OCD and other OCD-related factors, as well as a distinct factor specific to hair-pulling and skin-picking [27]. Our study found subjects with TD and comorbid trichotillomania appear more likely to have OCD, reaffirming the earlier results.

Another advantage of the TIC Genetics cohort is its geographical diversity. Subjects were from the USA, Europe, Israel, and South Korea, which permitted us to assess the potential population-specific genetic contribution to TD subtypes. Subjects from TIC Genetics centers in South Korea include 69 Asians (~8.4% of the total TIC Genetics cohort). Only about 10% of all Asian subjects with a diagnosis of TD also had a diagnosis of OCD, as compared to 40–60% observed in Caucasian subjects with TD diagnosis. A prior cross-national study indicated a lower prevalence of OCD among Asians compared to the worldwide prevalence [28]. Determining whether the apparent difference in rates of OCD comorbidity among regions is due to genetic variation among populations or other factors is an important question requiring further study. In addition, there are higher proportion of TD subjects with minimal or no NDD comorbidity subjects from the USA and Europe than from South Korea, suggesting ancestry/genetic background and/or assessment differences might account for some of these findings.

While our dataset is larger than most prior studies and has more parameters, this study still has several limitations. First, compared with phase 1, TIC Genetics phase 2 focused on recruiting simplex trios. However, we confirmed that this did not affect the unsupervised clustering because adding study phase as a parameter did not affect the clustering results. Second, as subjects with TD made up the vast majority of the subjects with tic disorders in the study, we only included subjects with TD to improve cluster stability. A larger dataset, including subjects with other tic disorder diagnoses, could permit better clustering despite the noisiness of the data and provide better grouping of tic disorder subtypes in addition to TD. Third, our dataset might not be large or varied enough to identify all the underlying subtypes with high accuracy. Larger data sets will enable us to effectively use machine learning and other methods (e.g., Variational Auto Encoders) for unsupervised clustering. Lastly, in addition to observations by the clinicians, a portion of the data is self-reported by the subject or their parents and might produce an uncertain level of errors. Even though the clinicians in the TIC Genetics study were trained to record the symptoms and diagnosis in a consistent manner, there may still be undetected variations between individual clinicians and sites. Despite all these limitations, the large and diverse TIC Genetics cohort enabled us to enhance the current understanding by identifying new and potentially relevant factors and subtypes.

## Future Direction

We are also exploring effective ways to include genetic data (e.g., SNP microarray and whole-exome sequencing data) available from the cohort to further identify subtypes. Another approach is to identify subtypes using genetic data and validate them using clinical data. The tools and methods we have evaluated and the workflow we developed here can also be used to evaluate hypotheses generated by domain experts. Ultimately, we plan to perform stratified analysis of genetic data based on the subtypes to identify new candidate genes and generate new insights into TD etiology, diagnosis, and treatment.

## Supplementary Material

Supplement 1

Supplement 2

Supplement 3

1

## Figures and Tables

**Table 1:** Comparison of K-Means (KM) and BHC Clusters

Kmclust	Kmsize+	BHCclust	BHCsize*	Pos. Correlation	Neg. Correlation
1	210	6	249	**ADHD**	Trichotillomania
				**OCD**	
				Male	
				ASD+	
				USA+	
				Tic Early Onset+	
2	40			**Trichotillomania**	
				USA	
		3	30	OCD+*	
		4	10		OCD*
3	289	5	250	**OCD**	**ADHD**
				Female	Trichotillomania
				Europe+	
4	229	2	206	South Korea+	**OCD**
				Tic Late Onset+	**ADHD**
					Trichotillomania
					ASD
5	97	1	120	**ADHD**	**OCD**
				Male+	TicSxCur
				ASD+	Trichotillomania

**Note:** Most statistically significant characteristics of each cluster are in bold

+ indicates of significance in Kmeans

* indicates of significance in BHC

The differences are only in cluster characteristics with low statistical significance
