# Supplementary material for: Identifying Distinct Tourette Disorder Subtypes using Clinical Data
Source: medRxiv. 2025 Nov 11:2025.11.09.25339700. Preprint. [Version 1] doi: 10.1101/2025.11.09.25339700 (PMC12642721; doi:10.1101/2025.11.09.25339700)
Supplement: 1 [file NIHPP2025.11.09.25339700V1-supplement-1.pdf]

medRxiv preprint doi: https://doi.org/10.1101/2025.11.09.25339700; this version posted November 11, 2025. The copyright holder for this preprint (which was not certified by peer review) is the author/funder, who has granted medRxiv a license to display the preprint in perpetuity.

It is made available under a CC-BY 4.0 International license .

Supplementary Tables

File: TD_SubTypes_SupplementaryTables.xlsx

Supplementary Table S1. TIC Genetics clinical data

Supplementary Table S2. Parameters used for clustering

Supplementary Table S3. Unsupervised clustering methods tested

Supplementary Table S4. R packages used in this study

Supplementary Table S5. Different data sets tested for identifying subtypes

Supplementary Table S6. K-Means Cluster Size and Characteristics

Supplementary Table S7. BHC Cluster Sizes and Characteristics

medRxiv preprint doi: https://doi.org/10.1101/2025.11.09.25339700; this version posted November 11, 2025. The copyright holder for this preprint (which was not certified by peer review) is the author/funder, who has granted medRxiv a license to display the preprint in perpetuity.

It is made available under a CC-BY 4.0 International license .

Supplementary Figures

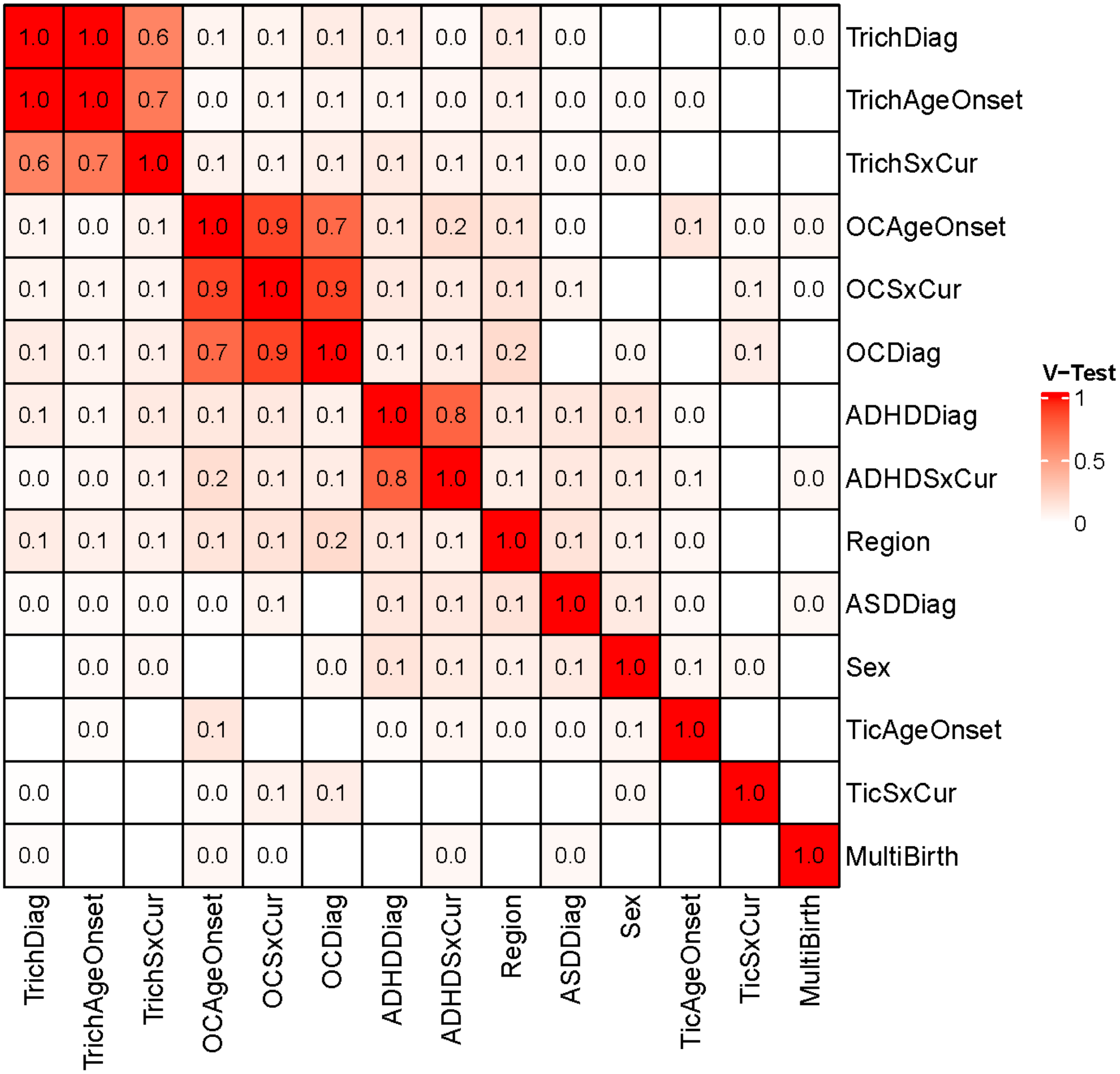

Supplementary Figure S1. Pairwise parameter correlation - Cramer's V

medRxiv preprint doi: https://doi.org/10.1101/2025.11.09.25339700; this version posted November 11, 2025. The copyright holder for this preprint (which was not certified by peer review) is the author/funder, who has granted medRxiv a license to display the preprint in perpetuity.

It is made available under a CC-BY 4.0 International license .

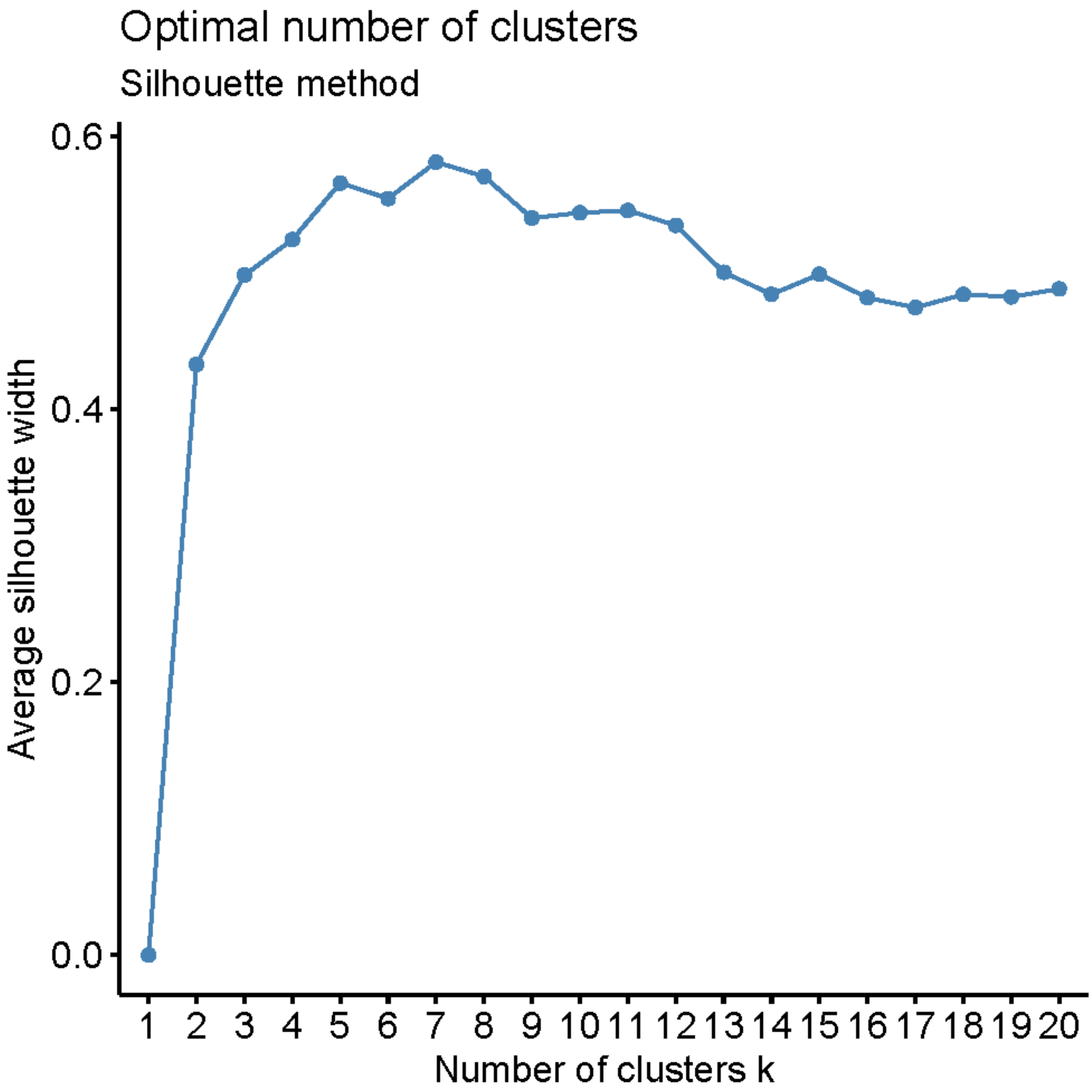

Supplementary Figure S2. Silhouette Plot for K-Means analysis with k=1 to 20

medRxiv preprint doi: https://doi.org/10.1101/2025.11.09.25339700; this version posted November 11, 2025. The copyright holder for this preprint (which was not certified by peer review) is the author/funder, who has granted medRxiv a license to display the preprint in perpetuity.

It is made available under a CC-BY 4.0 International license .

Supplementary Data

K-Means.xlsx – Category Description – Complete Results

BHC.xlsx – Category Description – Complete Results

High or low prevalence of a parameter represents a statistically significant difference between actual distribution phenotype in a cluster and compared to the expected distribution based on random sampling of our selected data set. R FactoMiner package [25] was used to generate the cluster characteristics and variable importance**.**
